# Supplementary material for: Supporting Occupational Physicians in the Implementation of Workers’ Health Surveillance: Development of an Intervention Using the Behavior Change Wheel Framework
Source: Int J Environ Res Public Health. 2021 Feb 17;18(4):1939. doi: 10.3390/ijerph18041939 (PMC7922522; doi:10.3390/ijerph18041939)
Supplement: Supplementary file 1 [file ijerph-18-01939-s001.zip › Supplementary Material A_FL.docx]

**Supplementary Material A: Results nominal group technique**

Participants: AB, CH, HM, FL

Round 1*:* In the first round, participants suggested to merge the components ‘the occupational physician (OP) considers implementing workers’ health surveillance (WHS) important (motivation) ’ and ‘the OP is motivated to implement WHS (motivation)’.

Round 2: In the second round, each participant selected the five COM-B components they considered most important to be included in the intervention. The COM-B components were ranked in order of importance by giving 5 points to the most important COM-B component, and 1 point for the least important component of the 5 selected COM-B components. The results are shown below.

|  | **Ranking** | | | |
| --- | --- | --- | --- | --- |
| **COM-B components** | AB | CH | HM | FL |
| 1. The OP has sufficient knowledge to initiate WHS at companies |  |  | 1 | 5 |
| 2. The OP has sufficient skills to initiate WHS at companies |  | 1 |  | 3 |
| 3. Occupational safety and health services have adopted an active policy towards the implementation of WHS | 2 |  |  |  |
| 4. There are sufficient (financial) possibilities to implement WHS (e.g. WHS is included in the contract with OP or the occupational health and safety service (OHS)). |  |  | 3 |  |
| 5. The employer has a positive attitude towards the implementation of WHS | 3 |  | 2 |  |
| 6. Workers have a positive attitude towards WHS |  |  |  |  |
| 7. The OP considers implementing WHS important + 9. The OP is motivated to implement WHS | 4 |  |  |  |
| 8. The OP considers initiating WHS as their task |  |  |  |  |
| 10. The OP has sufficient knowledge to determine the content of WHS |  | 5 | 5 |  |
| 11. The OP has sufficient knowledge to implement WHS (e.g. to conduct preventive consultations) | 5 | 3 | 4 | 4 |
| 12. The OP has sufficient skills to determine the content of the WHS |  | 4 |  |  |
| 13. The OP has sufficient skills to implement WHS (e.g. to conduct preventive consultations) |  | 2 |  | 2 |
| 14. There is sufficient time available for the OP to implement WHS |  |  |  |  |
| 15. There are sufficient possibilities to implement WHS | 1 |  |  |  |
| 16. There is a location available to implement WHS |  |  |  |  |
| 17. There are materials (medical equipment) available to implement WHS |  |  |  |  |
| 18. Workers consider the prevention of occupational diseases important (core objective 1) |  |  |  |  |
| 19. Employers consider the prevention of occupational diseases important (core objective 1) |  |  |  | 1 |
| 20. The OP considers implementing WHS as their task |  |  |  |  |

*Round 3:* In the third round, participants explained why they ranked these five COM-B components. The explanations of each participant are summarized below:

| ***Participant*** | ***Selected COM-B component*** | ***Explanation*** |
| --- | --- | --- |
| *AB* | 11. The OP has sufficient knowledge to implement WHS (e.g. to conduct preventive consultations) | Without knowledge, it is not possible to implement WHS |
| *AB* | 7+9. The OP considers implementing WHS important & The OP is motivated to implement WHS | The OP recognizes the importance of WHS, and will therefore implement WHS |
| *AB* | 5. The employer has a positive attitude towards the implementation of WHS | WHS will not be implemented without the employer's agreement. There is still much to be gained here |
| *AB* | 3. There is an active policy among occupational health and safety services towards the implementation of WHS | WHS will not be implemented without an active policy from the OHS. There is still much to be gained here. However, the OP is not directly influencing this. |
| *AB* | 15. There are sufficient possibilities to implement WHS | WHS will not be implemented without possibilities, but can this be influenced? |
|  |  |  |
| *CH* | 10. The OP has sufficient knowledge to determine the content of WHS | WHS starts with a good idea or content |
|  | 12. The OP has sufficient skills to determine the content of WHS | WHS starts with a good idea or content |
|  | 11. The OP has sufficient knowledge to implement WHS (e.g. to conduct preventive consultations) | Knowledge and skills are important. OPs in this study are already motivated to implement WHS |
|  | 13. The OP has sufficient skills to implement WHS (e.g. to conduct preventive consultations) | Skills are required to implement WHS |
|  | 2. The OP has sufficient skills to initiate WHS at companies | Some OPs do not know how to convince the employer, knowledge and skills have to be increased |
|  |  |  |
| *HM* | 10. The OP has sufficient knowledge to determine the content of WHS | It is essential to be able to design WHS, it therefore needed to increase knowledge and skills |
|  | 11. The OP has sufficient knowledge to implement WHS (e.g. to conduct preventive consultations) | It is essential to be able to design WHS, it therefore needed to increase knowledge and skills |
|  | 4. There are sufficient (financial) possibilities to implement WHS (e.g. WHS is included in the contract with OP or OHS) | WHS cannot be implemented without a contract |
|  | 5. The employer has a positive attitude towards the implementation of WHS | Without agreement of the employer, WHS cannot be implemented |
|  | 1. The OP has sufficient knowledge to initiate WHS | It is important to be able to explain to employers why WHS is important and needs to be implemented |
|  |  |  |
| *FL* | 1. The OP has sufficient knowledge to initiate WHS | Knowledge to initiate WHS is also necessary to be able to implement WHS |
|  | 11. The OP has sufficient knowledge to implement WHS (e.g. to conduct preventive consultations) | Knowledge to implement WHS is essential |
|  | 2. The OP has sufficient skills to put WHS on the agenda of employers | Skills must be taught to OPs to initiate and implement WHS |
|  | 13. The OP has sufficient skills to implement WHS (e.g. to conduct preventive consultations | Skills to implement WHS are essential |
|  | 19. Employers consider the prevention of occupational diseases important (core objective 1) | The attitude of the employer towards prevention of occupational diseases is important in the initiation of WHS by OPs |

*Round 4:* The five most important COM-B components were selected and ranked in the second ranking round.

|  | **Rankings** | | | |
| --- | --- | --- | --- | --- |
| **COM-B components** | AB | CH | HM | FL |
| 1. The OP has sufficient knowledge to initiate WHS | 2 | 2 |  | 5 |
| 2. The OP has sufficient skills to initiate WHS |  |  | 3 | 4 |
| 3. There is an active policy among occupational health and safety services towards the implementation of WHS | 1 |  |  |  |
| 4. There are sufficient (financial) possibilities to implement WHS (e.g. WHS is included in the contract with OP or OHS). |  |  |  |  |
| 5. The employer has a positive attitude towards the implementation of WHS | 4 |  | 1 | 3 |
| 10. The OP has sufficient knowledge to determine the content of WHS |  | 5 | 5 |  |
| 11. The OP has sufficient knowledge to implement WHS (e.g. to conduct preventive consultations) | 5 | 3 | 4 | 2 |
| 12. The OP has sufficient skills to determine the content of the WHS |  | 4 |  |  |
| 13. The OP has sufficient skills to implement WHS (e.g. to conduct preventive consultations) | 3 | 1 | 2 | 1 |

*Round 5:* In the fifth round, participants explained why they selected the five COM-B components, subsequently a group discussion was held about possibilities to include the COM-B components in the intervention program.

| ***Participant*** | ***Selected COM-B component*** | ***Explanation*** |
| --- | --- | --- |
| *AB* | 11. The OP has sufficient knowledge to implement WHS | Without sufficient knowledge, the OP cannot implement WHS |
| *AB* | 5. The employer has a positive attitude towards the implementation of WHS | WHS cannot be implemented without a positive attitude from the employer. There is still much to be gained |
| *AB* | 13. The OP has sufficient skills to implement WHS | Skills are essential |
| *AB* | 1+ 2. The OP has sufficient knowledge to initiate WHS + The OP has sufficient skills to initiate WHS | Merging 1 & 2, but they are also related to 5 |
| *AB* | 3. There is an active policy among occupational health and safety services to have companies implement WHS | WHS cannot be implemented without an active policy from the OHS |
|  |  |  |
| *CH* | 10. The OP has sufficient knowledge to determine the content of WHS | WHS starts with a good idea or content |
| *CH* | 12. The OP has sufficient skills to determine the content of the WHS | WHS starts with a good idea or content |
| *CH* | 11. The OP has sufficient knowledge to implement WHS | Knowledge and skills are important. OPs in this study are already motivated to implement WHS |
| *CH* | 1. The OP has sufficient knowledge to initiate WHS | Implementation makes more sense if you have properly determined the content and convinced the employer. |
| *CH* | 13 (The OP has sufficient skills to implement WHS) | Skills are required to implement WHS |
|  |  |  |
| *HM* | 10. The OP has sufficient knowledge to determine the content of WHS | It is essential to be able to design WHS, it is therefore needed to increase knowledge and skills |
| *HM* | 11. The OP has sufficient knowledge to implement WHS | It is essential to be able to design WHS, it therefore needed to increase knowledge and skills |
| *HM* | 2. The OP has sufficient skills to initiate WHS | Skills of OPs are easier to change, than attitude of employers |
| *HM* | 13. The OP has sufficient skills to implement WHS | This can be influenced in an intervention for OPs |
| *HM* | 5. The employer has a positive attitude towards the implementation of WHS | It is difficult to influence employers’ attitude directly by the OP |
|  |  |  |
| *FL* | 1 (The OP has sufficient knowledge to initiate WHS) | Knowledge and skills to initiate WHS is crucial in implementing WHS |
| *FL* | 2. The OP has sufficient skills to initiate WHS | Knowledge and skills to initiate WHS is crucial in implementing WHS |
| *FL* | 5. The employer has a positive attitude towards the implementation of WHS | The attitude of the employer can be a major barrier in the implementation of WHS |
| *FL* | 11. The OP has sufficient knowledge to implement WHS | Knowledge to implement WHS is essential |
| *FL* | 13. The OP has sufficient skills to implement WHS | Skills to implement WHS is essential |

*Main results group discussion*:

| **COM-B component** | **Summary of group discussion** |
| --- | --- |
| 11. The OP has sufficient knowledge to implement WHS (e.g. to conduct preventive consultations) (psychological capabilities) | Exercises with vignette studies to practice preventive consultations and giving advice  Blended learning to combine knowledge and skills, for example with online homework assignments |
| 10. The OP has sufficient knowledge to determine the content of WHS (psychological capabilities) | Sufficient skills are necessary to be able to actually determine the content of WHS  An overview of information needs to be developed to support OPs in determining the content of the WHS  Determining the content is strongly related to initiating WHS at companies  Determining the content of a WHS differs per subject / company / profession. You need to develop a general approach, of which 1 or 2 modules can be incorporated in an intervention |
| 1. The OP has sufficient knowledge to initiate WHS at companies (psychological capabilities) | Sufficient skills are also necessary to be able to actually initiate WHS  Knowledge needed to initiate WHS at companies in general is abstract  You need to know how to influence that one employer  Vignette studies with role play exercises must be done in face-to-face setting  Group discussion to discuss experiences of OPs  The OP must reach a level of expertise |
| 5. The employer has a positive attitude towards WHS (social opportunities) | The target behavior is that you can convince the employer of the utility and importance of WHS based on expertise |
| 13. The OP has sufficient skills to implement WHS (e.g. to conduct preventive consultations) (physical capabilities). | This can be included in a training, for example using role play exercises to practice the performance of preventive consultations with workers |

*Results final five selected COM-B components to include in the intervention program:*

1) The OP has sufficient knowledge to implement WHS (e.g. to conduct preventive consultations) (psychological capabilities)

2) The OP has sufficient knowledge to determine the content of WHS (psychological capabilities)

3) The OP has sufficient knowledge to initiate WHS at the employer (psychological capabilities)

4) The employer has a positive attitude towards the implementation of WHS (social opportunities)

5) The OP has sufficient skills to implement WHS (e.g. to conduct preventive consultations)(physical capabilities).
