# Supplementary material for: Supporting Occupational Physicians in the Implementation of Workers’ Health Surveillance: Development of an Intervention Using the Behavior Change Wheel Framework
Source: Int J Environ Res Public Health. 2021 Feb 17;18(4):1939. doi: 10.3390/ijerph18041939 (PMC7922522; doi:10.3390/ijerph18041939)
Supplement: Supplementary file 1 [file ijerph-18-01939-s001.zip › Supplementary Material B_FL.docx]

**Supplementary Material B:** BCW Step 5: Identify intervention functions using Affordability, Practicability, Effectiveness & cost-effectiveness, Acceptability, side effect/ unwanted consequences, and Equity (APEASE) criteria

| **BCW Intervention Functions** | **Affordability** | **Practicability** | **Effectiveness &**  **cost effectiveness** | **Acceptability** | **Side effects/ unwanted consequences** | **Equity** | **Comments** | **Decision** |
| --- | --- | --- | --- | --- | --- | --- | --- | --- |
| Education | ✓ | ✓ | ✓ | ✓ | **?** | **?** | Affordability: Access to educational programmes is affordable. Occupational physicians are required to attend a number of educational courses which often cost money [1].  Practicable: Increasing occupational physicians’ knowledge through educational programmes is practicable, it can be delivered to occupational physicians, as numerous of educational programs already exist [2, 3].  Effectiveness: Educational programmes for occupational physicians can be effective in increasing their knowledge [4].  Acceptable: Increasing occupational physicians’ knowledge through educational programmes is acceptable, numerous of previous educational programmes to increase occupational physicians’ knowledge already exist, as part of their in-service training requirement [2] [3] .  Side-effects: Educational programmes about workers’ health surveillance will probably not lead to unwanted side effects.  Equity: Increasing knowledge of occupational physicians concerning the initiation and implementation of workers’ health surveillance alone is not likely to lead to inequity. | Appropriate to include in the intervention |
| Training | ✓ | ✓ | ✓ | ✓ | **?** | **?** | Affordable: Access to training programmes are affordable. Occupational physicians are required to attend a number of in-service training programs [1] which often cost money. Practicable: Increasing occupational physicians’ skills through training programmes will cost a lot of time as increasing skills is more complex than increasing knowledge. As occupational physicians are in general very busy, a training program to increase skills may not be practicable. However, several training programmes to increase skills of occupational physicians already exists and have been used, and can be considered practical [2]. Effectiveness: A training program for occupational physicians did increase their self-efficacy regarding occupational health research and surveillance [5].  Acceptable: Increasing skills through training programmes is acceptable, there are numerous of programmes for occupational physicians to increase skills [2] Side-effects: Offering training programmes to increase skills to implement workers’ health surveillance is not likely to lead to unwanted side effects.  Equity: Offering training programmes to increase skills to implement workers’ health surveillance is not likely to lead inequity. | Appropriate to include in the intervention |
| Environmental Restructuring | **?** | 🗶 | **?** | 🗶 | **?** | **?** | Affordability: Changing the environment by making workers’ health surveillance more visible to employers or companies can be done by for example by distributing newsletters or flyers for employers. However, this may not be affordable for occupational physicians.  Practicability: A structured environment with regard to the initiation of workers’ health surveillance already exists through the policy of occupational health and safety services [6]. Changing this structured environment can be initiated by the occupational physician. However, the occupational health and safety service's policy remains in force to a certain extent, so that changing the environment is not entirely practicable. Effectiveness: The literature shows that there is some evidence on the effectiveness of environmental restructuring as an intervention to change behavior [7]. Specific evidence for the effectiveness of environmental restructuring in the context of occupational healthcare has nog been found yet.  Acceptability: Changing the employer's attitude towards workers’ health surveillance is not always acceptable. For example, interviews with occupational health and safety managers have shown that employers did not consider a policy of actively offering workers’ health surveillance to be acceptable [6]. Side-effects: It is unknown if changing the environment will have any unwanted effects on the health or safety of the parties involved. Equity: Changing the environment in order to change the attitude of the employer towards the implementation of workers’ health surveillance is unlikely to lead to inequity. | Not appropriate to include in the intervention |
| Restriction | 🗶 | 🗶 | 🗶 | 🗶 | 🗶 | 🗶 | Offering workers’ health surveillance is a legal obligation for the employer [8]. However, currently there is little enforcement on this law. In addition, the imposition of restrictions is outside our reach. | Not appropriate to include in the intervention |
| Enablement | ✓ | ✓ | ✓ | **?** | **?** | **?** | Affordability: A knowledge centre where occupational physicians, employers or workers could ask for advice about medical examinations for workers already exists [9], it is affordable.  Practicability: Setting up a helpdesk to support occupational physicians in overcoming barriers in the process of implementing workers’ health surveillance is practicable as there already is a knowledge center for medical examinations in workers, where questions about medical examinations for workers could be asked [9].  Acceptability: As a knowledge centre for medical examinations in workers already exists, it seems acceptable.  Effectiveness: As no research has been done about the effectiveness of the knowledge center, it is not known if enablement in the form of a helpdesk for occupational physicians is effective.  Side-effects: Risks of unwanted side effects of setting up a help desk for occupational physicians is likely to be minimal  Equity: Using a helpdesk or knowledge centre to enable occupational physicians to initiate workers’ health surveillance at companies is unlikely to lead to inequity. | Appropriate to include in the intervention |
| Modelling | **?** | **?** | **?** | **?** | **?** | **?** | Affordability: The costs of using modelling to improve the initiation of workers’ health surveillance by changing the attitude of employers towards workers’ health surveillance is unknown.  Practicability: It is unknown if it is practicable to use role models for occupational physicians.  Acceptability: It is unknown if it is acceptable to use role models for occupational physicians.  Effectiveness: For the context of occupational physicians no research has been done yet about modelling to change occupational physicians’ behavior. It was found that using local opinion leaders can be effective in promoting evidence-based practice [10].  Side-effects: Risk of unwanted side effects of using a role model is likely to be minimal  Equity: the effect of using a model on inequity is likely to be minimal | Not appropriate to include in the intervention |

1. Nederlandse Vereniging voor Arbeids- en Bedrijfsgeneeskunde. Bij-en nascholing. Accessed on 15 August 2020. Available from: <https://nvab-online.nl/de-bedrijfsarts/bij-en-nascholing>.

2. Netherlands School of Public and Occupational Health. **Bij-en nascholing** 2020. Accessed 6 August 2020. Available from: <https://www.nspoh.nl/bij-en-nascholing/>.

3. Curti S, Sauni R, Spreeuwers D, De Schryver A, Valenty M, Rivière S, et al. Interventions to increase the reporting of occupational diseases by physicians: a Cochrane systematic review. Occup Environ Med. 2016;73(5):353-4.

4. Smits PB, de Boer AG, Kuijer PP, Braam I, Spreeuwers D, Lenderink AF, et al. The effectiveness of an educational programme on occupational disease reporting. Occup Med (Lond). 2008;58(5):373-5.

5. Braeckman L, Venema A, Adigüzel van Zoelen S, Hermans L, De Ridder M, Ergör A, et al. Development and Evaluation of a Training Program on Occupational Health Research and Surveillance in Turkey. J Occup Environ Med. 2019;61(5):417-23.

6. Los FS, Hulshof CTJ, Sluiter JK. The view and policy of management of occupational health services on the performance of workers' health surveillance: a qualitative exploration. BMC Health Serv Res. 2019;19(1):473.

7. Chauhan BF, Jeyaraman MM, Mann AS, Lys J, Skidmore B, Sibley KM, et al. Behavior change interventions and policies influencing primary healthcare professionals' practice-an overview of reviews. Implement Sci. 2017;12(1):3.

8. Arbeidsomstandighedenwet 1998, artikel 18 1998. Accessed on 20 June 2020. Available from: <https://wetten.overheid.nl/BWBR0010346/2005-12-29#Hoofdstuk4>.

9. Kenniscentrum Medische Keuringen in Arbeid. Accessed on 6 August 2020. Available from: <https://www.amc.nl/web/specialismen/arbeidsgeneeskunde-1/arbeidsgeneeskunde-1/kenniscentrum-medische-keuringen-in-arbeid-kmka-1.htm>.

10. Flodgren G, O'Brien MA, Parmelli E, Grimshaw JM. Local opinion leaders: effects on professional practice and healthcare outcomes. Cochrane Database Syst Rev. 2019;6(6):Cd000125.
