# Supplementary material for: Supporting Occupational Physicians in the Implementation of Workers’ Health Surveillance: Development of an Intervention Using the Behavior Change Wheel Framework
Source: Int J Environ Res Public Health. 2021 Feb 17;18(4):1939. doi: 10.3390/ijerph18041939 (PMC7922522; doi:10.3390/ijerph18041939)
Supplement: Supplementary file 1 [file ijerph-18-01939-s001.zip › Supplementary Material C_FL.docx]

**Supplementary Material C:** BCW Step 7: Identify behavior change techniques using Affordability, Practicability, Effectiveness & cost-effectiveness, Acceptability, Side effects/ unwanted consequences, and Equity (APEASE) criteria

| **Intervention function** | **Potential behavior change techniques (BcT)** | **Elaboration on how the behavioral change technique could be used in the intervention program to change the COM-B components to achieve the desired behavior** | **Affordability** | **Practicability** | **Effectiveness &**  **cost effectiveness** | **Acceptability** | **Side effects/unwanted consequences** | **Equity** | **Comments APEASE** | **Decision** |
| --- | --- | --- | --- | --- | --- | --- | --- | --- | --- | --- |
| Education | **Information about social or environmental consequences** | Inform occupational physicians about social or environmental consequences of the implementation of workers’ health surveillance | **✓** | **✓** | **?** | **✓** | **?** | **?** | Affordability: Informing occupational physicians about social or environmental consequences of the implementation of workers’ health surveillance can be done during in-service educational programs or within guidelines. Providing information to occupational physicians to increase their knowledge has been done within numerous of existing educational programs, and guidelines, and is affordable for occupational physicians [1, 2]  Practicability: It is practicable to provide information about social or environmental consequences of the implementation of workers’ health surveillance. It can be provided within a training program or guideline, which has been done before [1, 2]  Effectiveness: No literature about the effectiveness of providing information about social or environmental consequences of behavior or actions to increase knowledge of occupational physicians was found.  Acceptability: It is acceptable to inform occupational physicians about social or environmental consequences of the implementation of workers’ health surveillance to increase knowledge.  Side-effects: It is unknown, but also unlikely that providing information about social or environmental consequences in an intervention program for occupational physicians will have unwanted side-effects Inequity: It is unknown, but also unlikely that providing information about social or environmental consequences to occupational physicians will lead to inequity. | Appropriate to include in the intervention |
| Education | **Information about health consequences** | Inform occupational physicians about health consequences for workers of different work-related risk factors, and on the possible effect of preventive interventions on the health of workers | **✓** | **✓** | **?** | **✓** | **?** | **?** | Affordability: Informing occupational physicians about health consequences of work-related risk factors and appropriate preventive interventions can be done during in-service educational programs or within guidelines. Providing information to occupational physicians to increase their knowledge has been done within numerous of existing educational programs, and guidelines, and is affordable for occupational physicians [1, 2]  Practicability: It is practicable to provide information about health consequences of work-related risk factors. It can be provided within a training program or guideline, which has been done before [1, 2]  Effectiveness: No literature about the effectiveness of providing information about consequences of behavior or actions to increase knowledge of occupational physicians was found.  Acceptability: It is acceptable to inform occupational physicians about health consequences of work-related risk factors to increase knowledge. Informing occupational physicians about consequences of risk factors for workers has been done before within guidelines, for example in guidelines for depression [3].  Side-effects: It is unknown, but also unlikely that providing information about consequences in an intervention program for occupational physicians will have unwanted side-effects Inequity: It is unknown, but also unlikely that providing information about consequences to occupational physicians will lead to inequity. | Appropriate to include in the intervention |
| Education | **Prompts/cues** | Using e-mails to remind occupational physicians about work-related health risks factors of different sectors, and the importance of workers’ health surveillance | **✓** | **?** | **?** | **?** | **?** | **?** | Affordability: To increase participation rate in research programmes and training programmes, we often send e-mail reminders to occupational physicians. Sending reminders can be done easily by e-mail and is affordable.  Practicability: To increase participation rate in research and training programmes, we often send reminders to occupational physicians. Sending reminders can be done easily by e-mail. However, not all occupational physicians did respond to e-mail reminders. Not all occupational physicians might have received the reminders, and practicability is unknown.  Effectiveness: Using computer generated reminders on paper for healthcare professionals to adhere to preventive guidelines can be effective [4]. However, effectiveness to remind occupational physicians about the importance of workers’ health surveillance in order to stimulate implementation is unknown.  Acceptability: In an earlier study we used e-mails to remind occupational physicians to complete their survey. However, we noticed that not all occupational physicians did complete the survey after our e-mail reminders. Acceptability of using prompts and cues is therefore unknown.  Side-effects: It is unknown, but also unlikely that using prompts and cues in an intervention program will have side-effects.  Inequity: It is unknown, but also unlikely that using prompts and cues to enable occupational physicians to initiate workers’ health surveillance will lead to inequity. | Not appropriate to include in the intervention |
| Education,  Training | **Feedback on behavior** | Monitoring and providing feedback on behavior of occupational physicians, for example during role play exercises in a training to improve occupational physicians’ knowledge and skills | ✓ | ✓ | **?** | ✓ | **?** | **?** | Affordability: Monitoring and providing feedback on behavior of occupational physicians during role play exercises has been done before within in-service training for occupational physicians. Numerous of such educational and training programmes for occupational physicians exist, and are affordable for occupational physicians [2].  Practicability: Monitoring and providing feedback on behavior of occupational physicians during role play exercises has been done before within in-service training programmes and is practicable. It can be organized within a training programme while participants play different roles to practice skills (e.g. employer, worker or occupational physician) [2].  Effectiveness/cost-effectiveness: Using feedback seemed to be an effective strategy to improve communication skills of physicians [5]. Effectiveness for using feedback to improve occupational physicians knowledge or skills regarding the implementation of workers’ health surveillance is unknown.  Acceptability: As monitoring and providing feedback on behavior of occupational physicians during role play has exercises been done within in-service training programmes, it is acceptable [2].  Side-effects: It is unknown, but also unlikely that using feedback in an intervention program for occupational physicians will have side-effects.  Inequity: It is unknown, but also unlikely that using feedback in a training program for occupational physicians will lead to inequity | Appropriate to include in the intervention |
| Education,  Training | **Feedback on outcomes of behavior** | Monitoring and providing feedback on outcomes of behavior can be done in an intervention program in which occupational physicians report the results of implementation of workers’ health surveillance (e.g. preventive interventions implemented), and receive feedback to the results of the implementation, for example by e-mail | **?** | ✓ | 🗶 | **?** | **?** | **?** | Affordability: It is unknown if it is affordable to provide feedback on outcomes of behavior of occupational physicians through e-mail.  Practicability: Providing feedback on outcomes of behavior through e-mail is practicable as we assume that all occupational physicians know how to use e-mail  Effectiveness/cost-effectiveness: The study of Lenderink et al., (2010) showed that providing stage-matched feedback to occupational diseases on outcomes reporting occupational diseases was not effective in changing their behavior (e.g. reporting of occupational disease) [6].  Acceptability: It is unknown if it is acceptable for occupational physicians to report their results on the implementation of workers’ health surveillance and receive feedback.  Side-effects: It is unknown, but also unlikely that using feedback on outcomes of behavior in an intervention program for occupational physicians will have side-effects.  Inequity: It is unknown, but also unlikely that using feedback on outcomes of behavior of occupational physicians will lead to inequity | Not appropriate to include in the intervention |
| Education, training | **Self-monitoring of behavior** | Self-monitoring of occupational physicians’ knowledge and skills to determine the content of workers’ health surveillance, initiate and implement workers’ health surveillance using questionnaires | **✓** | **?** | **?** | **?** | **?** | **?** | Affordability: Using surveys for occupational physicians to self-monitor their knowledge and skills concerning the initiation and implementation of workers’ health surveillance has been done in our cohort study. This was affordable. Practicability: We did not evaluate occupational physicians’ experience of self-monitoring their knowledge and skills within our cohort study. Not all occupational physicians completed the survey, it is unknown if it is practicable to use self-monitoring of behavior. Effectiveness: As we might assume that monitoring behavior will make occupational physicians more aware of their behavior, it may be effective. It has not been shown that self-monitoring is an effective strategy in increasing knowledge and skills, and it therefore unknown.  Acceptability: As we already monitor knowledge and skills of occupational physicians through a survey study, is seems acceptable. However, for the second wave of our cohort study, not all occupational physicians completed the survey, indicating that self-monitoring of knowledge and skills might not be acceptable for occupational physicians.  Side-effects: It is unknown, but also unlikely that using self-monitoring in an intervention program for occupational physicians will have side-effects.  Inequity: It is unknown, but also unlikely that using self-monitoring will lead to inequity. | Not appropriate to include in the intervention |

| Training | **Demonstration of the behavior** | Providing an example of a  preventive consultation with a worker, or conversation with an employer to initiate workers’ health surveillance during a training | **✓** | **✓** | **?** | **✓** | **?** | **?** | Affordability: Demonstration of consultations with workers about the results of workers’ health surveillance can be done by actors during a training program for occupational physicians. It has been done before in training programmes for occupational physicians, and is affordable [2].  Practicability: Demonstration of consultations with workers is practicable as it has been done before during in-service training for occupational physicians. A seven-step protocol for the consultation with workers is also available for occupational physicians [7].  Effectiveness/cost-effectiveness: Evidence for using demonstration of behavior to increase skills of occupational physicians to implement workers’ health surveillance is lacking, it is unknown.  Acceptability: In earlier in-service training programs for occupational physicians to increase skills to implement workers’ health surveillance, we used demonstration of consultation with workers, using the 7-step protocol [7]. This was found to be acceptable.  Side-effects: It is unknown, but also unlikely that using demonstration of the behavior in an intervention program for occupational physicians will have side-effects. Inequity: It is unknown, but also unlikely that using demonstration of behavior in a training program for occupational physicians will lead to inequity. | Appropriate to include in the intervention |
| --- | --- | --- | --- | --- | --- | --- | --- | --- | --- | --- |
| Training | **Instructions on how to perform behavior** | Providing instructions on how to initiate workers’ health surveillance, determine the content of workers’ health surveillance or conduct preventive consultations with workers, for example providing instructions in a guidance document, or during a training program. | **✓** | **✓** | **?** | **✓** | **?** | **?** | Affordability: Instructions for occupational physicians about how to determine the content of workers’ health surveillance, and how to initiate workers’ health surveillance are affordable as it can be part of an in-service training, of which numerous already exist [2]. Moreover, instructions on paper for occupational physicians, for example within a guidance document also exist and are affordable for occupational physicians [1, 8]. Practicability: Providing instructions on for example how to conduct consultations with workers is practicable as numerous of in-service training programs, and instructions on paper already exist [7, 9].  Effectiveness/cost-effectiveness: Evidence for the effectiveness of using instructions to increase skills of occupational physicians is lacking, it is unknown.  Acceptability: As numerous of training programs with instructions and guidelines to increase skills of occupational physicians already exist, it is acceptable [8] [2].  Side-effects: It is unknown, but also unlikely that using instructions in an intervention program for occupational physicians will have side-effects.  Inequity: It is unknown, but also unlikely that using instructions in a training program for occupational physicians will lead to inequity. | Appropriate to include in the intervention |
| Training | **Behavioral practice/rehearsal** | Behavioral practice of skills to initiate workers’ health surveillance, determine the content of workers’ health surveillance and conduct preventive consultations with workers can be done with role play exercises. For example within a training program for occupational physicians | ✓ | ✓ | ? | ✓ | **?** | **?** | Affordability: Rehearsal of behaviors of occupational physicians can be done within a training program, vignette studies and role play exercises can be used to practice skills to determine the content of workers’ health surveillance, initiate and implement workers’ health surveillance. This is affordable as it can be done within an in-service training for occupational physicians, of which numerous already exist [2].  Practicability: Rehearsal of initiating and determining the content of workers’ health surveillance, and conducting preventive consultation with workers can be done within a training program, vignette studies and role play exercises can be used. This is practicable as other training programs for occupational physicians already had rehearsal included [2].  Effectiveness: Rehearsal of skills has shown to be an important component for possible effectiveness of teaching strategies to improve communication skills of physicians [5]. We assume that using rehearsal is also effective for occupational physicians. However, evidence is lacking.  Acceptability: As rehearsal of skills has been used in training programmes for physicians and occupational physicians before, it is acceptable [2].  Side-effects: It is unknown, but also unlikely that using rehearsal in an intervention program for occupational physicians will have side-effects Inequity: It is unknown, but also unlikely that using rehearsal in a training program for occupational physicians will lead to inequity | Appropriate to include in the intervention |

| Enablement | **Social support** | Enabling occupational physicians to initiate workers’ health surveillance at companies by arranging social support in the form of feedback and advise for occupational physicians to overcome barriers they encounter (e.g. resistance of employers) For example by setting up a knowledge centre or helpdesk | ✓ | ✓ | **?** | **?** | **?** | **?** | Affordability: As there already is a knowledge centre, in which occupational physicians can ask for advice or support about initiation of workers’ health surveillance, it is affordable to provide social support for occupational physicians to enable them to initiate workers’ health surveillance at companies [10].  Practicability: It seems practicable to provide social support to occupational physicians to enable them to initiate workers’ health surveillance at companies, as a knowledge centre to provide support to occupational physicians already exist [10].  Effectiveness/cost-effectiveness: Research among physicians in specialization training shows that they considered social support important to achieve learning goals [11]. However, effectiveness of social support for occupational physicians to enable them in the initiation of WHS has not been investigated yet.  Acceptability: As a similar knowledge centre already exist, it seems acceptable [10]. However, as not all occupational physicians do consult the knowledge centre to ask for support, it is unknown if it is acceptable.  Side-effects: It is unknown, but also unlikely that using social support in an intervention program for occupational physicians will have side-effects.  Inequity: It is unknown, but also unlikely that using social support in a training program for occupational physicians will lead to inequity. | Not appropriate to include in the intervention |
| --- | --- | --- | --- | --- | --- | --- | --- | --- | --- | --- |
| Enablement | **Goal setting** | Setting a behavioral goal and/or outcome goal for occupational physicians concerning the initiation of workers’ health surveillance with employers | **✓** | **✓** | **?** | **?** | **?** | **?** | Affordability: It is affordable for occupational physicians to set goals concerning the initiation of workers’ health surveillance at companies. It can be integrated within in an in-service training program, of which numerous already exist [2].  Practicability: Goal setting for occupational physicians concerning the initiation of workers’ health surveillance is practicable when it can be done as part of a training program for occupational physicians.  Effectiveness: Goal setting has shown to be an important component of an effective self-regulated learning strategy of physicians [12]. We can assume that reviewing goals is also effective in education for occupational physicians. However, evidence is still lacking.  Acceptability: It is unknown if it is acceptable for occupational physicians to set goals concerning their behavior of implementation of workers’ health surveillance as part of an intervention program. Side-effects: It is unknown, but also unlikely that using goal setting in an intervention program for occupational physicians will have side-effects Inequity: It is unknown, but also unlikely that using goal setting in a training program for occupational physicians will lead to inequity | Appropriate to include in the intervention |
| Enablement | **Adding objects to the environment** | Adding objects to the environment, for example an app for occupational physicians with tips and tricks for the initiation of workers’ health surveillance | **?** | **?** | **?** | **?** | **?** | **?** | Affordability: It is unknown if it is affordable for occupational physicians to use apps with tips and tricks to initiate workers’ health surveillance at companies Practicability: It is unknown if using an app with tips and tricks for the initiation of workers’ health surveillance is practicable Effectiveness: It has been found that using digital education can be effective in increasing knowledge of health professionals [13]. However, evidence for the effectiveness of adding objects to the environment to change behavior of occupational physicians has not been found.  Acceptability: It is unknown if it is acceptable for occupational physicians to add objects to the environment  Side-effects: It is unknown, but also unlikely that adding objects to the environment in an intervention program for occupational physicians will have side-effects Inequity: It is unknown, but also unlikely that adding objects to the environment in a training program for occupational physicians will lead to inequity | Not appropriate to include in the intervention |
| Enablement | **Problem solving** | Discussing occupational physicians’ experiences with the initiation of workers’ health surveillance, and possible solutions.  Subsequently, an overview can be created for occupational physicians to use in the initiation of workers’ health surveillance at companies or employers | ✓ | 🗶 | ✓ | ✓ | **?** | **?** | Affordability: Discussing and creating an overview of experiences and possible solutions of barriers for the initiation of workers’ health surveillance is affordable as it can be organized easily during in-service training [2].  Practicability: Organizing a meeting with occupational physicians to discuss barriers and develop solutions seems practicable as it can be done as an in-service training for occupational physicians, of which numerous already exist [2]. However, direct solutions for resistance of employers towards workers’ health surveillance is very difficult to create, and not practicable.  Effectiveness/cost-effectiveness: A study among occupational physicians showed that discussing barriers and possible solutions in small-learning peer sessions is effective in contributing to knowledge and skills of occupational physicians’ relating to adherence of a guideline [14].  Acceptability: Problem solving is acceptable within an intervention program for occupational physicians, as it can be easily organized as an in-service training program, which is required for occupational physicians [2]. Moreover, occupational physicians in the training program of Joosen et al.,2018 agreed that the training program was effective [14].  Side-effects: It is unknown, but also unlikely that problem solving within an intervention program for occupational physicians will have side-effects Inequity: It is unknown, but also unlikely that problem solving in a training program for occupational physicians will lead to inequity | Not appropriate to include in the intervention |
| Enablement | **Action planning (implementation strategy)** | Guiding occupational physicians in creating a detailed planning of the initiation of workers’ health surveillance, including action planning | **✓** | **✓** | **?** | **?** | **?** | **?** | Affordability: Action planning can be done within a training session for occupational physicians, and is affordable as several training programs for occupational physicians exist as in-service training [2].  Practicability: Using action planning for the initiation of workers’ health surveillance is practicable for occupational physicians, as they can create the action plan during an in-service training.  Effectiveness/cost-effectiveness: A study investigating the use of action planning to improve nurses’ hand hygiene shows that action planning is useful, and may be effective [15]. However, for the context of occupational physicians, this is unknown.  Acceptability: The acceptability of using action planning to enable occupational physicians to initiate workers’ health surveillance at companies is unknown.  Side-effects: It is unknown, but also unlikely that using action planning within an intervention program for occupational physicians will have side-effects.  Inequity: It is unknown, but also unlikely that using action planning in a training program for occupational physicians will lead to inequity. | Appropriate to include in the intervention |
| Enablement | **Restructuring the physical environment** | Restructuring the physical environment by adding information about the importance of workers’ health surveillance on posters and flyers, visible for workers and employers to change the attitude of employers towards workers’ health surveillance | **?** | **?** | **?** | **?** | **?** | **?** | Affordability: It is not known if restructuring the physical environment for occupational physicians is affordable.  Practicability: Restructuring the physical environment by using posters and flyers might not be practicable as each occupational physician need distribute a few posters and flyers. However, this is unknown.  Effectiveness: No literature about restructuring the environment to change behavior of occupational physicians concerning the initiation of workers’ health surveillance has been found. Effectiveness is unknown.  Acceptability: It is not known if restructuring the physical environment for the initiation of workers’ health surveillance is acceptable.  Side-effects: It is unknown, but also unlikely that restructuring the physical environment in an intervention program for occupational physicians will have side-effects.  Inequity: It is unknown, but also unlikely that restructuring the physical environment in a training program for occupational physicians will lead to inequity | Not appropriate to include in the intervention |
| Enablement | **Review behavioral and outcome goals** | Reviewing behavioral or outcome goals of occupational physicians concerning the initiation of workers’ health surveillance at employers, for example at the end of training sessions | ✓ | ✓ | **?** | **?** | **?** | **?** | Affordability: When goals are set during an in-service training, reviewing goals can also be done as part of a in-service training program. Numerous of in-service training programmes for occupational physicians already exist, which is affordable for occupational physicians to participate in [2].  Practicability: When goals are set during a training program, reviewing goals can be done in a follow-up meeting.  Effectiveness/cost-effectiveness: The effectiveness of reviewing goals as a behavior change technique for occupational physicians is unknown.  Acceptability: It is unknown if reviewing goals of occupational physicians is acceptable. Side-effects: It is unknown, but also unlikely that reviewing goals in an intervention program for occupational physicians will have side-effects.  Inequity: It is unknown, but also unlikely that reviewing goals in a training program for occupational physicians will lead to inequity. | Appropriate to include in the intervention |

1. Nederlandse Vereniging voor Arbeids- en Bedrijfsgeneeskunde. NVAB-richtlijnen . Accessed on 6 August 2020. Available from: <https://nvab-online.nl/richtlijnen/richtlijnen-NVAB>.

2. Netherlands School for Public and Occupational Health. **Bij-en nascholing** 2020. Accessed on 6 August 2020. Available from: <https://www.nspoh.nl/bij-en-nascholing/>.

3. Nederlandse Vereniging voor Arbeids- en Bedrijfsgeneeskunde. Richtlijn depressie voor bedrijfsartsen en verzekeringsartsen 2016. Accessed on 6 August 2020. Available from: <https://nvab-online.nl/sites/default/files/bestanden-webpaginas/RL_Depressie_26-09-2016_2.pdf>.

4. Arditi C, Rège-Walther M, Durieux P, Burnand B. Computer-generated reminders delivered on paper to healthcare professionals: effects on professional practice and healthcare outcomes. Cochrane Database Syst Rev. 2017;7(7):Cd001175.

5. Berkhof M, van Rijssen HJ, Schellart AJ, Anema JR, van der Beek AJ. Effective training strategies for teaching communication skills to physicians: an overview of systematic reviews. Patient Educ Couns. 2011;84(2):152-62.

6. Lenderink AF, Spreeuwers D, van der Klink JJ, van Dijk FJ. Information and feedback to improve occupational physicians' reporting of occupational diseases: a randomised controlled trial. Int Arch Occup Environ Health. 2010;83(4):381-8.

7. Ketelaar SM, Nieuwenhuijsen K, Gärtner FR, Bolier L, Smeets O, Sluiter JK. Mental Vitality @ Work: The effectiveness of a mental module for workers' health surveillance for nurses and allied health professionals, comparing two approaches in a cluster-randomised controlled trial. Int Arch Occup Environ Health. 2014;87(5):527-38.

8. Sluiter J, Weel ANH, Hulshof C. Leidraad preventief medisch onderzoek van werkenden 2013. Accessed on 6 June 2020. Available from: <https://nvab-online.nl/sites/default/files/bestanden-webpaginas/Leidraad_PMO5c1b.pdf>.

9. Nederlandse Vereniging voor Arbeids- en Bedrijfsgeneeskunde. Bij-en nascholing. Accessed on 15 August 2020. Available from: <https://nvab-online.nl/de-bedrijfsarts/bij-en-nascholing>.

10. Kenniscentrum Medische Keuringen in Arbeid. Available from: <https://www.amc.nl/web/specialismen/arbeidsgeneeskunde-1/arbeidsgeneeskunde-1/kenniscentrum-medische-keuringen-in-arbeid-kmka-1.htm>.

11. Mikkola L, Suutala E, Parviainen H. Social support in the workplace for physicians in specialization training. Med Educ Online. 2018;23(1):1435114.

12. van Houten-Schat MA, Berkhout JJ, van Dijk N, Endedijk MD, Jaarsma ADC, Diemers AD. Self-regulated learning in the clinical context: a systematic review. Med Educ. 2018;52(10):1008-15.

13. Tudor Car L, Soong A, Kyaw BM, Chua KL, Low-Beer N, Majeed A. Health professions digital education on clinical practice guidelines: a systematic review by Digital Health Education collaboration. BMC Med. 2019;17(1):139.

14. Joosen MCW, van Beurden KM, Rebergen DS, Loo M, Terluin B, van Weeghel J, et al. Effectiveness of a tailored implementation strategy to improve adherence to a guideline on mental health problems in occupational health care. BMC Health Serv Res. 2019;19(1):281.

15. Erasmus V, Kuperus MN, Richardus JH, Vos MC, Oenema A, van Beeck EF. Improving hand hygiene behaviour of nurses using action planning: a pilot study in the intensive care unit and surgical ward. J Hosp Infect. 2010;76(2):161-4.
