# Supplementary material for: Supporting Occupational Physicians in the Implementation of Workers’ Health Surveillance: Development of an Intervention Using the Behavior Change Wheel Framework
Source: Int J Environ Res Public Health. 2021 Feb 17;18(4):1939. doi: 10.3390/ijerph18041939 (PMC7922522; doi:10.3390/ijerph18041939)
Supplement: Supplementary file 1 [file ijerph-18-01939-s001.zip › Supplementary Material D_FL.docx]

**Supplementary Material D: BCW Step 8: Identify modes of delivery using Affordability, Practicability, Effectiveness & cost-effectiveness, Acceptability, Side effects/ unwanted consequences, and Equity (APEASE) criteria**

| **Mode of delivery** | | **Elaboration for the intervention program/BCT** | **Affordability** | **Practicability** | **Effectiveness &**  **cost effectiveness** | **Acceptability** | **Side effects/unwanted consequences** | **Equity** | **Comments APEASE** | **Decision** |
| --- | --- | --- | --- | --- | --- | --- | --- | --- | --- | --- |
| **Face-to-face** | **Individual** | Individual lectures for occupational physicians, for example about consequences of work-related health risks for workers, about instructions on how to initiate workers’ health surveillance at companies, or about how to conduct preventive consultations with workers | 🗶 | ✓ | **?** | **?** | **?** | **?** | Affordability: It would not be affordable to deliver individual lectures to occupational physicians  Practicability: Delivering individual lectures, for example about work-related risk factors for the health of workers would be practicable, as the lecture can be delivered to the participant.  Effectiveness: It is unknown if it is effective to deliver lectures individually to occupational physicians.  Acceptability: It is not known if it is acceptable for occupational physicians to participate in individual lectures as part of an intervention program.  Side-effects: It is unknown, but unlikely that there will be side-effects as a result of individual lectures for occupational physicians.  Equity: We don’t expect much effect on equity from individual lectures | Not appropriate |
|  | **Group** | Group lectures for occupational physicians, for example about the work-related health risks for workers | ✓ | ✓ | ✓ | ✓ | **?** | **?** | Affordability: Face-to-face lectures for a group of occupational physicians about work-related risk factors for the health of workers would be affordable. Different group lectures to inform occupational physicians already exists and are affordable, for example Heijermans lectures [1].  Practicability: Delivering face-to-face lectures about work-related health risks for workers for a group of occupational physicians is practicable as such lectures already exist for occupational physicians [1].  Effectiveness: It is effective to deliver face-to-face group lectures to occupational physicians. In a study about lecture-based learning versus problem based learning in post graduate medical education for occupational physicians, it was found that knowledge increased in both groups [2].  Acceptability: In the study of Smits et al., 2003 it was found that satisfaction was higher in the lecture-based group than in the problem-based education group [2]. It is thus acceptable to use lectures to inform occupational physicians.  Side-effects: It is unlikely that there will be side-effects from face-to face group lectures for occupational physicians about work-related risk factors.  Equity: We don’t expect much effect on equity from group lectures | Appropriate |
|  |  | Group training for occupational physicians on the implementation of workers’ health surveillance. For example with role play exercises to practice a conversation with the employer about the initiation of workers’ health surveillance | **✓** | **✓** | **✓** | **✓** | **?** | **?** | Affordability: It is affordable to organize group training sessions with occupational physicians, as many of such training programs already exist [3].  Practicability: It is practicable to organize group training sessions with occupational physicians, as many of such training programs already exist [3].  Effectiveness: Different training programs for occupational physicians to increase skills exist, and are effective. For example the study of Braeckman et al., (2019) found an increase in level of self-efficacy in occupational physicians after a training program on occupational health research and surveillance [4].  Acceptability: It is acceptable to organize group training sessions with occupational physicians, as many of such training programs already exist [3].  Side-effects: It is unknown but also unlikely that group training sessions will have side-effects.  Equity: We don’t expect much effect on equity from a group training for occupational physicians. | Appropriate |
|  |  | Organizing work place visits for occupational physicians to inform them about consequences of work-related health risks | **?** | **?** | **✓** | **?** | **?** | **?** | Affordability: Occupational physicians do regularly visit work places to investigate the risks for the health of workers. However, it is unknown if this will be affordable as part of an educational program about workers’ health surveillance for occupational physicians.  Practicability: It is unknown if it is practicable to include workplace visits in an educational program to inform occupational physicians about work-related risk factors.  Effectiveness: The study of Braeckman et al.,2009 among medical students showed that workplace visits can be effectively applied to increase awareness of students about risk factors and preventive measures as part of their education in occupational health [5].  Acceptability: It is unknown if it is acceptable for occupational physicians to include workplace visits in an intervention program regarding the implementation of workers’ health surveillance.  Side-effects: It is unknown but unlikely that work place visits as part of an educational program for occupational physicians will lead to side-effects.  Equity: We don’t expect much effect on equity from workplace visits | Not appropriate |
|  |  | Group discussions with real employers or workers to discuss the implementation of workers’ health surveillance | **?** | **✓** | **✓** | **?** | **?** | **?** | Affordability: It is unknown if group sessions with both occupational physicians and employers or workers to discuss the implementation of workers’ health surveillance is affordable as we don’t know if real employers and workers are willing to participate in such sessions  Practicability: It would be practicable to organize sessions with employers or workers and occupational physicians as they can learn from each other and generate a realistic view of the conversations  Effectiveness: The study of Brackman et al., (2014) showed that discussing case studies with real patients is an effective method for medical students to achieve learning objectives in occupational medicine [6].  Acceptability: It is unknown if it would be acceptable for occupational physicians to participate in group sessions with real employers or workers.  Side-effects: It is unknown but unlikely that participation in group sessions with real employers or workers will have side-effects for occupational physicians.  Equity: We don’t expect much effect on equity from group discussions with real employers or workers | Not appropriate |
|  |  |  |  |  |  |  |  |  |  |  |
| **Distance**  **Population level** | **Broadcast media**   - **TV** - **Radio** | A TV or radio commercial to increase knowledge of occupational physicians to implement workers’ health surveillance, for example describing the work related risk factors for the health of workers | **?** | 🗶 | **?** | **?** | **?** | **?** | Affordability: It is unknown if it would be affordable to use radio or TV commercials to inform occupational physicians about work-related risk factors for the health of workers  Practicability: It would not be practicable to use radio or TV commercials as it will not be focused on occupational physicians alone  Effectiveness: It is unknown if it is effective to use radio or TV commercials to inform occupational physicians about work-related health risk factors.  Acceptability: It is unknown if it would be acceptable to use radio or TV commercials to inform occupational physicians about work-related health risk factors.  Side-effects: It is unknown but unlikely that radio or TV commercials to inform occupational physicians about work-related risk factors will have side-effects  Equity: We don’t expect much effect on equity from TV or radio commercials | Not appropriate |
|  | **Outdoor media**   - **Billboard** - **Poster** | Info graphic on outdoor media showing information about the implementation of workers’ health’ surveillance, for example consequences of work-related risk factors, or instructions on how to conduct preventive consultations | **?** | 🗶 | **?** | **?** | **?** | **?** | Affordability: It is unknown if it would be affordable to use posters or bill boards to inform occupational physicians about workers’ health surveillance.  Practicability: Not much information can be provided on a poster or bill board, this may be not practicable.  Effectiveness: It is unknown if it would be effective to use posters or billboards to inform occupational physicians about the implementation of workers’ health surveillance  Acceptability: It is unknown if it would be acceptable to use posters or bill boards to inform occupational physicians about the implementation of workers’ health surveillance  Side-effects: It is unknown but unlikely that posters or bill boards about workers’ health surveillance for occupational physicians will have side-effects.  Equity: We don’t expect much effect on equity from posters or billboards | Not appropriate |
|  | **Print media**   - **Newspaper** - **Other written material** | Using articles in journals for occupational physicians to educate them in the implementation of workers’ health surveillance, for example instructions on how to initiate workers’ health surveillance at companies | **✓** | 🗶 | **?** | **✓** | **?** | **?** | Affordability: It would be affordable to publish an article in a journal for occupational physicians to educate them on how to initiate workers’ health surveillance, as many informative articles are published for occupational physicians already.  Practicability: It would be practicable to publish an article in a journal for occupational physicians. However, this article cannot cover all detailed information,as articles cannot be too long. This is not very practicable.  Effectiveness: It is unknown if it is effective to use informative articles in journals for occupational physicians to increase their knowledge for the implementation of WHS.  Acceptability: It would be acceptable to publish an article in a journal for occupational physicians, as many informative articles are published already.  Side-effects: It is unknown but unlikely that informative articles in journals for occupational physicians will have side-effects.  Equity: We don’t expect much effect on equity from informative articles in journals for occupational physicians. | Not appropriate |
|  | **Digital media**   - **Internet (website)** - **Mobile phone app** |  |  |  |  |  |  |  |  |  |
|  |  | E-learning about the implementation of workers’ health surveillance, for example with informative text and videos about work-related risk factors for the health of workers, or demonstration videos of preventive consultations with workers | **✓** | **✓** | **✓** | **✓** | **?** | **?** | Affordability: It would be affordable to use informative text and videos to inform occupational physicians about work-related health risk factors  Practicability: Using an e-learning with informative text and videos about work-related risk factors is practicable. Different e-learnings for occupational physicians already exist [7].  Effectiveness: Using videos is as effective as using traditional lectures in increasing knowledge of medical students [8]. Moreover, it was found that e-learnings are effective in increasing knowledge of occupational physicians [9].  Acceptability: Informative text and videos can be included in an e-learning module for occupational physicians about work-related health risk factors for workers. E-learnings for occupational physicians appear to be acceptable as the majority of the occupational physicians was able to finish an e-learning course and considered the course as clear and understandable [9].  Side-effects: It is unknown but also unlikely that online informative videos for occupational physicians about work-related health risk factors will have side-effects.  Equity: We don’t expect much effect on equity from informative videos in an e-learning | Appropriate |
|  |  | Online platforms about workers’ health surveillance in which occupational physicians can describe their experiences with the initiation or implementation of workers’ health surveillance, and other occupational physicians can provide feedback on the described behavior | **?** | **?** | **?** | **✓** | **?** | **?** | Affordability: It is unknown if it is affordable to use an online platform in which occupational physicians can describe their experiences with the implementation of workers’ health surveillance, and can provide feedback on experiences of other occupational physicians  Practicability: It is unknown if it is practicable to use an online platform in which occupational physicians can describe their experiences with the implementation of workers’ health surveillance and can provide feedback on experiences of other occupational physicians  Effectiveness: It is unknown if it is effective to use an online discussion platform to increase knowledge and skills of occupational physicians to implement workers’ health surveillance  Acceptability: The study of Kolb et al., (2009) found that an online platform with case-based e-learnings with feedback from teacher in occupational medicine was acceptable for participants [10].  Side-effects: It is unknown, but also unlikely that there will be any unwanted side-effects from an educational discussion platform for occupational physicians  Equity: We don’t expect much effect on equity from a discussion platform for occupational physicians | Not appropriate |
| **Distance**  **Individual level** | **Phone**   - **Mobile help line** - **Mobile phone text** | Providing a mobile phone help line for occupational physicians to ask for information on how to initiate workers’ health surveillance at companies | 🗶 | 🗶 | **?** | **?** | **?** | **?** | Affordability: It is not affordable to use a help line for occupational physicians as there has to be someone available for this help line every day  Practicability: A help line for occupational physicians will not provide them with an overview of information, and is therefore not practicable.  Effectiveness: It is unknown if it is effective to use a help line for occupational physicians with information about the implementation of workers’ health surveillance  Acceptability: It is unknown if it would be acceptable for occupational physicians to use a mobile phone help line to ask for information about the implementation workers’ health surveillance  Side-effects: It is unknown but unlikely that a mobile phone help line for occupational physicians will have side-effects  Equity: We don’t expect much effect on equity from a mobile phone help line | Not appropriate |
|  | **Individually accessed computer program** | An individually accessed computer program with informative text about workers’ health, and videos with explanation or demonstration on how to implement workers’ health surveillance | 🗶 | 🗶 | **?** | **?** | **?** | **?** | Affordability: It is not affordable to use an individually accessed computer program.  Practicability: It would not be practicable to use an individually accessed computer program as the content cannot be easily adjusted to new developments in occupational healthcare.  Effectiveness: It is unknown if an individually accessed computer program will increase knowledge of occupational physicians.  Acceptability: It is unknown if it is acceptable for occupational physicians to install a computer program.  Side-effects: It is unknown but also unlikely that a computer program for occupational physicians will have side-effects  Equity: We don’t expect much effect on equity from an individual accessed computer program for occupational physicians. | Not appropriate |

1. Nederlands Centrum voor Beroepsziekten. Heijermanslezingen. Accessed on 6 September 2020. Available from: <https://www.beroepsziekten.nl/ncvb/heijermanslezingen>.

2. Smits PB, de Buisonjé CD, Verbeek JH, van Dijk FJ, Metz JC, ten Cate OJ. Problem-based learning versus lecture-based learning in postgraduate medical education. Scand J Work Environ Health. 2003;29(4):280-7.

3. Netherlands School of Public and Occupational Health. **Bij-en nascholing** 2020. Accessed on 6 August 2020. Available from: <https://www.nspoh.nl/bij-en-nascholing/>.

4. Braeckman L, Venema A, Adigüzel van Zoelen S, Hermans L, De Ridder M, Ergör A, et al. Development and Evaluation of a Training Program on Occupational Health Research and Surveillance in Turkey. J Occup Environ Med. 2019;61(5):417-23.

5. Braeckman L, Bekaert M, Cobbaut L, De Ridder M, Glazemakers J, Kiss P. Workplace visits versus case studies in undergraduate occupational medicine teaching. J Occup Environ Med. 2009;51(12):1455-9.

6. Braeckman L, t Kint L, Bekaert M, Cobbaut L, Janssens H. Comparison of two case-based learning conditions with real patients in teaching occupational medicine. Med Teach. 2014;36(4):340-6.

7. Netherlands School of Public and Occupational Health. E-learning . Accessed on 20 August 2020. Available from: <https://www.nspoh.nl/e-learning/>.

8. Yiu SHM, Spacek AM, Pageau PG, Woo MYC, Curtis Lee A, Frank JR. Dissecting the Contemporary Clerkship: Theory-based Educational Trial of Videos Versus Lectures in Medical Student Education. AEM Educ Train. 2020;4(1):10-7.

9. Hugenholtz NI, Sluiter JK, van Dijk FJ, Nieuwenhuijsen K. EBM E-learning: Feasible and Effective for Occupational Physicians in Different Countries. Saf Health Work. 2012;3(3):199-208.

10. Kolb S, Wengenroth L, Hege I, Praml G, Nowak D, Cantineau J, et al. Case based e-learning in occupational medicine--a European approach. J Occup Environ Med. 2009;51(6):647-53.
